# Animal-borne soundscape logger as a system for edge classification of sound sources and data transmission for monitoring near-real-time underwater soundscape

**DOI:** 10.1038/s41598-024-56439-x

**Published:** 2024-03-16

**Authors:** Takuji Noda, Takuya Koizumi, Naoto Yukitake, Daisuke Yamamoto, Tetsuro Nakaizumi, Kotaro Tanaka, Junichi Okuyama, Kotaro Ichikawa, Takeshi Hara

**Affiliations:** 1 Biologging Solutions Inc., Kyoto, Japan; 2 Japan Fisheries Science and Technology Association, Tokyo, Japan; 3 Ocean Policy Research Institute of the Sasakawa Peace Foundation, Tokyo, Japan; 4 Fisheries Technology Institute, Japan Fisheries Research and Education Agency, Okinawa, Japan; 5 Field Science Education and Research Center, Kyoto University, Kyoto, Japan

**Keywords:** Soundscape, Biologging, Edge classification, Deep learning, Ocean noise, Electrical and electronic engineering, Marine biology, Physical oceanography, Animal migration, Biodiversity

## Abstract

The underwater environment is filled with various sounds, with its soundscape composed of biological, geographical, and anthropological sounds. Our work focused on developing a novel method to observe and classify these sounds, enriching our understanding of the underwater ecosystem. We constructed a biologging system allowing near-real-time observation of underwater soundscapes. Utilizing deep-learning-based edge processing, this system classifies the sources of sounds, and upon the tagged animal surfacing, it transmits positional data, results of sound source classification, and sensor readings such as depth and temperature. To test the system, we attached the logger to sea turtles (*Chelonia mydas*) and collected data through a cellular network. The data provided information on the location-specific sounds detected by the sea turtles, suggesting the possibility to infer the distribution of specific species of organisms over time. The data showed that not only biological sounds but also geographical and anthropological sounds can be classified, highlighting the potential for conducting multi-point and long-term observations to monitor the distribution patterns of various sound sources. This system, which can be considered an autonomous mobile platform for oceanographic observations, including soundscapes, has significant potential to enhance our understanding of acoustic diversity.

## Introduction

The underwater environment, representing the world’s most extensive habitat, encompasses a diverse array of sounds produced by various organisms (biophony). In addition to biophony, this environment comprises geological sounds, such as sounds caused by wind-driven waves and rain (geophony), and sounds generated by human activities like shipping and underwater construction (anthrophony). Soundscapes featuring this assortment of sounds, defined as “environmental sounds in terms of their spatial, temporal, and frequency attributes and the types of sources contributing to the sound field” (ISO 18405:2017, ISO, 2017)^1,2^.

Within diverse marine soundscapes, the frequency ranges, sound pressure level (SPL), and particle motion (PM) components exhibit vast geographical variation, reflecting the complex dynamics of natural and anthropogenic influences^2,3^. Natural sounds such as waves and rain (geophony) contribute to the soundscape, with waves in the 200–2000 Hz range^4^ and rain creating sounds in the 15–20 kHz range^5^. Human activities such as shipping, underwater construction, and offshore wind farm operations create artificial sounds that span a wide frequency spectrum, which can be categorized into low-frequency sounds ranging from 10 to 500 Hz, medium-frequency sounds spanning 500 Hz–25 kHz, and high-frequency sounds exceeding 25 kHz^6^. Marine life, ranging from large whales to small invertebrates, produces sounds spanning from infrasonic (< 20 Hz) to ultrasonic frequencies (> 20 kHz), serving crucial ecological functions such as navigation, communication, and foraging^3,7,8^. However, most of these biological sounds are emitted between 10 Hz and 20 kHz^7–9^, indicating the crucial importance of monitoring this frequency range for understanding the acoustic environment of these organisms.

In recent years, noise issues in the ocean, and conflicts between marine organisms and human communities have gained increasing attention^2^. Anthropogenic noise, for instance, can interfere with marine animals’ natural auditory signal processing, causing “masking”^10,11^, which narrows their communication space^12^. Furthermore, the increases in cyclones and ocean heat waves, driven by climate change, can also alter biophony^2^. Specifically, coral reef degradation associated with these events can dramatically change the surrounding soundscape, with community composition shifts reflected in reduced overall acoustic energy and altered complexity and diversity of reef soundscapes^13^. In light of these challenges, passive acoustic monitoring (PAM), a remote sensing technique using a hydrophone to capture the underwater world's soundscape, has become increasingly valuable. PAM serves as a noninvasive method for assessing biodiversity through analysis of the soundscape and plays a crucial role in observing underwater environmental changes and their impacts on marine life^6,7,9,14^.

Real-time identification and dissemination of underwater soundscape information could help raise public awareness of aquatic noise issues and biodiversity stewardship. Soundscape information could also help monitor and manage biodiversity and coral reef healthiness in Marine Protected Areas (MPAs) and for monitoring illegal fishing or navigation. Instead of merely summarizing the underwater soundscape using traditional acoustic indicators like sound pressure, complexity, or entropy^15^, a more practical approach may be to focus on identifying specific sound sources. This is essential because these conventional acoustic indicators, developed primarily for terrestrial research, may not effectively represent the biodiversity of the underwater environment^16,17^. Providing more detailed sound classification results promotes understanding of diverse sound sources and enables more informed actions to address conflicts between organisms and human activities.

A potential system for real-time soundscape monitoring could involve deploying buoys equipped with acoustic recording, sound classification functions, and satellite or cellular network transmission capabilities^18^. However, fixed-point observation limits its spatial coverage, and particularly in coastal areas, installation of recorders and buoys is often challenging due to the overlapping with fisheries and maritime activities. To complement this limitation, mobile platforms such as marine gliders and drifting recorders have been introduced^19,20^. Although those methods extend spatial coverage, they are not always suitable in shallow waters and coral reef areas because of their complex geographical characteristics. Given the high biodiversity in coastal areas, an alternative monitoring platform is required for effective environmental assessment.

Biologging has recently emerged as an effective method for oceanographic observations, treating animals like sea turtles and seals as autonomous moving platforms. Since 2002, instrumented animals have contributed over 650,000 conductivity-temperature-depth (CTD) profiles to the Global Ocean Observing System (GOOS) network via the Global Telecommunication System (GTS)^21^. Animal-based observation is particularly effective in polar and coastal regions^22–24^, where Argo floats and ship-based observations are difficult. Thus, biologging may serve as an efficient real-time soundscape monitoring system, filling the gaps in areas where traditional methods such as Argo floats and ship-based observations are challenging. However, no reported system utilizes animals for real-time monitoring of underwater soundscapes.

In the realm of real-time monitoring of underwater soundscapes through biologging, a crucial aspect is the implementation of sound classification. Confronting the challenge of transmitting raw audio data in real-time, primarily due to bandwidth and storage constraints of biologging devices^25,26^, it becomes imperative to process sounds directly on the device, a technique known as edge processing^27^. This method is significant as it allows only essential, classified data to be transmitted, reducing the volume of data and enhancing the functionality of biologging devices. Sound classification has evolved from feature-based methods^28^, machine learning^29^, and more recently, deep learning approaches^30–32^, which has been identified as the most accurate for underwater sound classification^33^. The strength of deep learning lies in its ability to autonomously extract complex features from raw audio data and accurately identify intricate patterns^34^. Deep learning uses layered neural networks for pattern recognition and decision-making with minimal human input^35^, efficiently processing large data volumes, ideal for real-time biologging in edge processing.

Before the practical implementation of biologging for soundscape observation, which would include the development of applications for notifications and establishing field operation systems, it is essential to first develop the monitoring devices themselves. This study aimed to develop a biologging device (i.e., a soundscape logger) to classify sound sources and transmit the classification results in near-real time. Specifically, the objectives of this study were (1) to design the soundscape logger and (2) to collect data in the field using the logger developed as a demonstration. This study conducted experiments on the green turtle *Chelonia mydas*, as the model species, that come ashore to lay eggs in the coral reef area of Ishigaki Islands, Okinawa, Japan. The choice of sea turtles as the model species was motivated by several factors. Primarily, their behavior of surfacing for breathing allows for near-real-time observation without the need for physical device retrieval^36^. Furthermore, we specifically focused on sea turtles during the nesting season. These turtles typically engage in multiple nesting landings within a single season^37^, providing opportunities for direct logger recovery, crucial for evaluating the prototype. Additionally, concentrating on the coastal coral reef habitat, a region of rich biodiversity and human activity^38^, aligns with our aim to assess equipment in diverse acoustic environments. Moreover, sea turtles, which migrate post-breeding^37^ can be utilized in biologging studies for environmental monitoring, as their movements provide valuable ancillary environmental data^21–23^. While the present study emphasized the coastal regions during the nesting period for the purpose of direct device retrieval, the broad habitat range of sea turtles suggests their potential applicability in expansive oceanic monitoring in future research initiatives.

## Materials and methods

### Concept of the animal-borne soundscape logger

**Figure 1:**
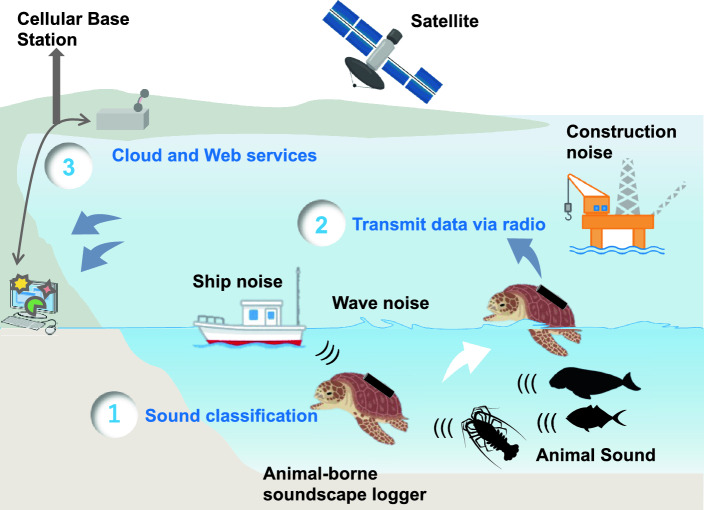
Conceptual diagram of the animal-borne soundscape logger. The logger, attached to a sea turtle, (1) records and classifies sound while recording environmental data such as depth, temperature, salinity, and dissolved oxygen, as well as behavior data, including acceleration and geomagnetism, while the turtle is underwater. (2) Upon surfacing, the logger records the turtle’s position using GPS and transmits sound classification results and sensor measurement data to the cloud via cellular or satellite communication. (3) The data transmitted to the cloud is stored on a server and can be accessed through a web-based system.

The logger has a sound classification function, environmental measurement function, behavior measurement function, GPS capabilities, and a data transmission function (Fig. 1). Precisely when attached to a surfacing animals such as sea turtles seals, and sharks, the logger records and classifies sound data, measures environmental data such as depth, temperature, salinity, and dissolved oxygen, and behavior data, including acceleration and geomagnetism, while the animal is underwater. Upon surfacing, the turtle's position is recorded using GPS, and the sound classification results and sensor measurement data are transmitted to the cloud (online server) via radio waves.

Two methods of data transmission from the sea are considered: cellular network communication and satellite communication, such as Argos^25^ and Iridium^39^. While satellite communication employs radio waves to transmit data via satellites, cellular networks use radio waves for ground-based tower-to-device communication. The data sent to the cloud can be accessed through a web-based system.

In this study, we primarily utilized cellular networks available in the coastal regions of Japan for monitoring sea turtles nesting on the shore. Recognizing, however, that marine animals often venture into offshore areas beyond the reach of cellular networks, we also developed a logger variant capable of Argos satellite communication. This variant, which uses radio waves for satellite data transmission, is designed for use in open ocean regions and will be independently validated in future studies. Importantly, the Argos satellite communication system enables data collection even during the post-nesting migration of sea turtles, ensuring continuous monitoring as they move into open oceanic waters. Although Argos communication was not employed in this specific study, our approach integrates both cellular and satellite communication methods—both utilizing radio waves—to showcase a comprehensive strategy for marine animal monitoring.

The classified sound types obtained, in this case, can be combined with location data to illustrate where and what kinds of sounds were observed, essentially creating a “soundscape map” and a histogram of sound types in that marine area. While location data is only recorded when the animals, such as sea turtles, surface for breathing^40^, a time-lag occurs since sound classification data are collected underwater. To address this challenge, sounds classified within a certain time before and after the location data acquisition are considered to be associated with that specific location, creating an integrated dataset of sound types and positions. While the current methodology of our study does not encompass the use of inertial navigation data from sensors like speed and direction sensors for underwater positioning^41^, integrating such data could potentially enable more precise correlations between classified sounds and their specific locations. Furthermore, by combining this position-associated sound type data with environmental conditions like depth, temperature, salinity, and dissolved oxygen, as well as behavior information derived from metrics like acceleration, it becomes possible to simultaneously understand under what conditions these sounds were observed.

### Development of the deep learning analysis of sound classification

#### Target underwater sounds

Given the prevalence of biological, environmental, and anthropogenic sounds below 20 kHz^2,3,42^, this study focused on sounds ranging from 60 Hz to 20 kHz, excluding clicks from small cetaceans above 20 kHz. This study selected 52 sound classes consisting of biological, geographical, and anthropogenic sounds as target sounds to classify them at three levels of detail. The levels range from primary classification (Level 1) of biophony, geophony, or anthrophony, to more detailed classification (Level 2), such as distinguishing fish sounds from marine mammal sounds within biophony or vessel sounds from construction sounds within anthrophony. The most detailed classification (Level 3) specifies animal species, call types within the same species, and vessel types (ferry, tanker, pleasure boat, etc.). A library of the 52 sounds (51 sound classes plus background noise) for constructing a deep learning model was compiled, with details of the 52 sounds provided in Supplementary Table S1. The recording methodology for the target sounds is detailed in Tanaka et al. (unpublished data), and a database of sound sources is being developed in our project. To briefly explain, the sound source library was created using video and acoustic recorders deployed in the field at Sekisei Lagoon around Yaeyama Islands and other marine areas and in tanks housing individual organisms. Previously recorded data in other research were also incorporated. It should be noted that for distant sounds, direct observation of the emitting species is not always possible. However, the calls of whales and dolphins, for example, have been reported in various studies^43,44^. Experts in marine mammal acoustics have identified these sounds based on spectrograms, observations, habitat ranges, and characteristics of sounds from prior research.

For fish sounds, recordings were made in the field with stationary cameras. As not all species known to produce sounds were captured on video, the observed organisms were captured and their sound production was confirmed in tanks, thereby identifying the sounds. When recording in tanks, we ensured that only the intended organism was present, allowing us to confidently attribute the sound to that organism. Recording techniques to avoid tank reverberation involve considerations of tank size, microphone placement, and environment setup, with details available in Akamatsu et al.^45^.

This study did not account for overlaps in sound sources. The library excludes recordings with overlapping sounds, and a short time window of 2 s was adopted (details of sound processing are described later) to minimize potential overlap. It is important to note that the dataset includes a variety of sound types, ranging from continuous sounds such as those from ships to short, pulse-like sounds typical of fish. This diversity is considered in the analysis despite the exclusion of overlapping sounds.

Tank experiments were conducted at Churaumi Aquarium, Hakkeijima Sea Paradise, and in tanks established at a fishery cooperative on Ishigaki Island. Acoustic recorders employed included AUSOMS Mini, and AUSOMS ver. 3 (Aqua Sound Inc., Kobe, Japan), and LoggLaw CAM (Biologging Solutions Inc., Kyoto, Japan). A GoPro Hero 7 video camera (GoPro Inc., San Mateo, CA, USA) was concurrently used alongside the acoustic recorder, except when using the LoggLaw CAM, which recorded both underwater sound and video as a single unit.

#### Sound classification algorithm

In this study, we focused on sound sources ranging from 60 Hz to 20 kHz, with the objective of classifying these sound sources using loggers attached to sea turtles. The hydrophone used featured an omnidirectional design with a flat sensitivity response from 1 Hz to 100 kHz. Audio data was captured at a sampling rate of 44.1 kHz and a 16-bit resolution. Detailed specifications and setup information are provided in the “Developed soundscape logger” section. This equipment facilitated the development of a specialized algorithm for sound source classification within the specified frequency range.

**Figure 2:**
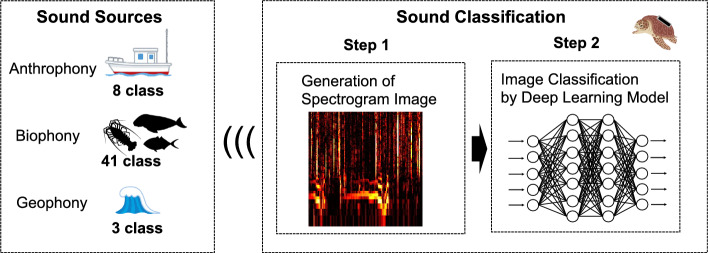
Overview of the sound classification algorithm. The sound classification algorithm follows a simple two-step process: pre-processing the raw sound to generate a spectrogram image and subsequently using a deep learning model to classify the spectrogram image.

Given adequate computational resources, complex deep learning algorithms, such as YOLO^46^ and Mask R-CNN^47^ for object detection or audio separation algorithms like TasNet^48^, can be utilized. However, for the practical implementation of a biologging device, the device size should be minimized to facilitate animal attachment, and the device should operate solely on battery power without an external supply. Thus, the classification algorithm needs to be computationally efficient. After conducting extensive preliminary trials, we employed a simple two-step process (Fig. 2) for sound source classification. Specifically, the process involved pre-processing the raw sound to generate a spectrogram image, followed by image classification using a deep learning model. This two-step method aligns with the approach described by Tanaka et al. (unpublished data), and detailed algorithmic background is described in that study. Here, we further optimized the signal processing for the biologging device, which is described in detail in the subsequent sections.

#### Selection of processing unit

While the logger relies exclusively on battery power, the battery size must be constrained to facilitate attachment to a sea turtle. Moreover, as deep learning models typically undergo retraining and updating upon data revision, loggers should be designed to accommodate continuous model updates. Potential computing units for executing deep learning models include CPUs (microcontrollers), GPUs (Graphics Processing Units), FPGAs (Field-Programmable Gate Arrays), and specialized AI chips (e.g., https://www.coral.ai/). Upon comparison, CPUs were deemed appropriate for this application due to their relatively low power consumption and ease of model updating, despite the drawback of extended processing times.

We opted for a two-step approach for the sound classification algorithm consisting of sound pre-processing and deep learning. To maximize power efficiency in a biologging device, executing the classification process using the microcontroller’s internal memory and RAM (Random Access Memory) rather than relying on external memory or RAM is preferable. Employing external memory or RAM necessitates power and time for data input/output, expands the physical space required for component installation, and increases standby power consumption. Thus, we selected a microcontroller with ample internal memory and RAM to run deep learning models in-house and with relatively low power consumption (STM32 H7 series from STMicroelectronics Inc, Geneva, Switzerland, featuring up to 1 Mbyte of internal Flash memory and 512 Kbytes of RAM as a stand-alone computation storage area). This microcontroller was among those with extensive internal memory and RAM available when the logger was designed (December 2021).

#### Implementation method in microcontroller

Google’s TensorFlow Lite (https://www.tensorflow.org/lite) is a framework for executing deep learning on microcontrollers and other embedded devices. Among TensorFlow Lite’s models is MobileNet^49^, an efficient image classification model lightweight enough to implement on mobile devices with constrained computing resources and battery power. A preliminary study proposed two enhanced versions of MobileNet, ver. 2, and ver. 3, following the initial ver. 1 release. However, since ver. 2 and ver. 3 required excessive memory and RAM for the selected microcontroller, rendering them unsuitable for STM32 H7 series microcontroller implementation, we opted for ver. 1.

MobileNet employs two key variables: input image size and width multiplier. The width multiplier is a hyperparameter that scales down the number of channels in each layer, effectively controlling the network’s ‘width’. This reduction in channels decreases the model's computational cost and size, enhancing efficiency, especially for mobile or resource-limited environments. Similarly, a smaller input image size requires less memory and RAM for processing. It’s important to note that while reducing the width multiplier (with a maximum of 1.0) and the input image size lessens memory and computational demands, it may impact the model’s accuracy. However, to perform image classification using deep learning on a microcontroller, the memory and RAM consumed by the entire process, from spectrogram calculation to actual classification by the deep learning model, must fit within the microcontroller's memory and RAM capacity.

**Figure 3:**
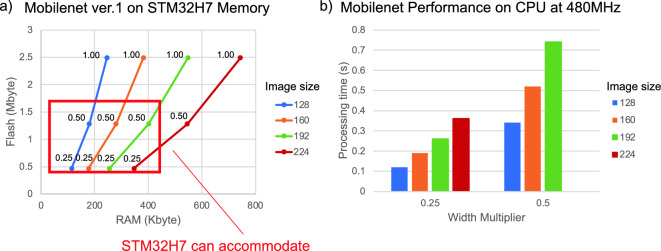
Flash and RAM size and processing time simulation. (**a**) Flash size (Mbyte) and RAM size (Kbyte) consumed during microcontroller execution for varying input image sizes (128 × 128, 160 × 160, 192 × 192, and 224 × 224 pixels, indicated by colors) and different width multipliers (0.25, 0.50, and 1.00, indicated by the number next to the point on the polyline) of MobileNet ver. 1. This data, reflecting the resource consumptions, is a recalculation in our environment, referencing the content described in the following URL (https://github.com/EEESlab/mobilenet_v1_stm32_cmsis_nn). (**b**) Processing time (Y-axis) for running MobileNet ver. 1 on the STM32H7 microcontroller at its maximum clock speed of 480 MHz under the conditions of input image size (128 × 128, 160 × 160, 192 × 192, and 224 × 224 pixels, indicated by colors) and width multiplier (x-axis) compatible with the STM32H7 microcontroller.

We utilized X-Cube AI from STMicroelectronics Inc. (https://www.st.com/en/embedded-software/x-cube-ai.html) to verify which settings can be implemented in the microcontroller when the input size and width multiplier of the image are altered. The models employed for verification were sample models downloaded from TensorFlow Hub (https://tfhub.dev/). All models used were fully quantized 8-bit models. Validation results indicated that width multipliers of 0.25 and 0.50 could be implemented for image sizes 128, 160, and 192, while a width multiplier of 0.25 could be implemented for image size 224 (Fig. 3a).

Additionally, for continuous sound classification, one image is generated for every 2 s of sound data, as detailed in subsequent sections, requiring classification to be completed within a maximum of 2 s for timely processing. Therefore, we evaluated the time consumption of MobileNet conditions with varying width multipliers and input image sizes, executable on the previously mentioned microcontroller, using IAR Embedded Workbench (IAR Systems, Uppsala, Sweden). Our findings indicate that with the STM32 H7 microcontroller operating at 480 MHz, its maximum clock speed, processing can be completed within one second (Fig. 3b).

Although the verification mentioned above has identified the MobileNet image size and width multiplier suitable for microcontroller execution, pre-processing must also be considered. For instance, in a PC with ample computing resources, the following processing has proven effective in preliminary studies for pre-processing (Tanaka et al., unpublished data). For sound data pre-processing, a spectrogram is computed using 1024 points for the short-time Fourier transform (STFT) with a 0.9 overlap ratio for 10 s of input data. The calculations are performed using Fast Fourier Transform (FFT). Notably, the frequency axis of the spectrogram is represented on a logarithmic scale, enhancing the detail in lower frequencies and more effectively covering the broad frequency range, compared to a linear scale. To reduce stationary background noise, a median filter is applied directly to the 10-s spectrogram data. In this process, the median value is determined for each data point, effectively smoothing the signal and reducing noise. This technique is commonly used in signal processing to preserve sharp signal features while removing noise, making it particularly suitable for enhancing the clarity of spectrogram data. Subsequently, for sound classification, a 2-s time window is used to analyze the audio data. This window slides forward by 1 s for each step, overlapping with half of the previous window. As a result, for every 10 s of audio data, we generate nine overlapping segments, each 2 s in length. From each of these segments, an image is created, resulting in a total of nine images for each 10-s interval. However, performing the same pre-processing on a microcontroller was challenging due to memory and RAM size limitations. Thus, we investigated how altering the input sound’s time window length and the FFT overlap ratio would impact the system’s feasibility on a microcontroller. Moreover, to minimize the memory and RAM requirements for pre-processing within the microcontroller, we opted to compute in single precision (32-bit floating point format) instead of double precision (64-bit floating point format). Single precision offers sufficient accuracy for our needs while using less computational resources compared to double precision, which provides higher numerical accuracy but at a greater memory cost.

Longer input time window lengths for the sound data, such as 10 s, make it more probable for the median filter to separate the target sound from stationary background noise for sounds occurring within a relatively short duration (e.g., within 1 s). In our study, we chose a 0.5 FFT overlap ratio to address the limitations of the microcontroller’s RAM. The window length for each FFT bin is fixed at 1024, maintaining a consistent temporal resolution. While a higher overlap ratio like 0.9 would spread the energy over more bins, leading to a smoother appearance in the spectrogram, it significantly increases the data volume. This increase in data volume, as demonstrated in Supplementary Table S2, is not feasible within our hardware constraints. Therefore, we established a 5-s input sound time window length and a 0.5 FFT overlap ratio for the logger, striking a balance between the requirements of our data analysis and the memory capacity of the hardware.

Based on the abovementioned validation, we further simulated memory and RAM consumption from sound data input to pre-processing and MobileNet execution. We found that the deep learning model can be executed on a microcontroller with a 5-s window, a 0.5 FFT overlap ratio, a 192 × 192 image size, and a 0.25 width multiplier. In this setup, the 192 × 192 image size corresponds to a 2-s audio spectrogram, where each pixel on the horizontal axis represents 0.015 s (2 s ÷ 192 pixels). Vertically, the range of 60 Hz–20 kHz is displayed on a logarithmic scale across 192 pixels, with each pixel representing a different frequency range that expands at higher frequencies and contracts at lower frequencies. Additionally, the use of square images aligns with the MobileNet model’s design for square inputs, allowing us to fully utilize its architecture and pre-trained weights. This pre-processing algorithm was employed to process a library of 52 sound sources (Supplementary Table S1) and generate spectrogram images for constructing a deep learning model. It is important to note that while the study categorized these sounds into three levels of detail, the algorithm was trained exclusively on the most detailed classification, Level 3. This level includes the specification of animal species, call types within the same species, and vessel types, etc. By focusing on this granular level, the model inherently learns to distinguish the broader categories of Level 1 (biophony, geophony, anthrophony) and Level 2 (subcategories within biophony and anthrophony). Therefore, classification of Level 3 allows for an implicit classification of the higher-level categories (Level 1 and Level 2), ensuring a comprehensive classification across all levels of detail.

#### Evaluation of sound classification performance

A sound dataset comprising a total of 5999 samples was used in this study, with the detailed number of samples for each class provided in Supplement 1. Of the total sound data, 70% was utilized as training data and 30% as test data. The training data was subsequently used to create a TensorFlow model for classifying soundscape image data using MobileNet ver. 1 transfer learning. The model was converted to a TensorFlowLite model and fully quantized, making all weights and activation outputs 8-bit integer data. The pre-processing was programmed in C (https://www.gnu.org/software/gnu-c-manual/gnu-c-manual.html) on a PC, and the same code was run on the microcontroller to ensure consistency in the image data calculated by the pre-processing. The program for creating the TensorFlowLite model was developed on Google Colab (https://colab.research.google.com/) using Python (https://docs.python.org/3/reference/index.html). The accuracy of the classification results was evaluated using the following metrics.

$$Precision = \frac{TP}{{TP + FP}}$$

$$Recall = \frac{TP}{{TP + FN}}$$

$$F{ - }measure = \frac{2Recall \cdot Precision}{{Recall + Precision}}$$

$$Accuracy = \frac{TP + TN}{{TP + FP + FN + TN}}$$

TP stands for True Positive, FP for False Positive, TN for True Negative, and FN for False Negative. The precision represents the percentage of predicted positive data that are positive. The recall represents the percentage of truly positive data correctly predicted as positive. The F-measure, calculated as the harmonic mean, is an evaluation metric to assess the balance between Precision and Recall. Additionally, machine learning algorithms commonly provide an accuracy metric, calculated as the ratio of correct predictions to the total number of data points. In image classification, accuracy is assessed in two scenarios: when the model’s most probable classification label (Top1) matches the correct answer and when the correct answer is among the top five most probable classification labels predicted by the model (Top5). Therefore, accuracy was evaluated in both cases to provide a comprehensive performance evaluation. To present the results of multiple classification classes, a Confusion Matrix was employed.

#### Data transmission

In the realm of mobile telecommunications, 4G, particularly LTE (Long Term Evolution), is globally prevalent^50^. LTE is a standard for wireless broadband communication, providing enhanced speeds and network capacity. Our research primarily concentrated on LTE-M (Long Term Evolution for Machines)^51^, a novel communication standard recently introduced for the Internet of Things (IoT), also known as CAT M1 (Category M1). LTE-M is known for its enhanced data communication efficiency and power-saving capabilities compared to LTE. The LTE-M standard specifies a maximum data rate of 1 Mbps, and for this study, we utilized a communication module capable of transmitting a maximum of 560 bytes per packet. Data transmission is accomplished through LTE-M, along with MQTT (Message Queuing Telemetry Transport)^52^, a lightweight messaging protocol. The transmitted information is then processed by Amazon Web Services (AWS), a cloud computing platform provided by Amazon.com, Inc. (Seattle, USA). The data first passes through the AWS IoT Hub for initial handling, is decoded using AWS Lambda, a serverless computing service, and ultimately stored in Amazon DynamoDB, a NoSQL database service, all of which are part of the comprehensive suite of services offered by AWS. We selected a cellular network provided by NTT Docomo Inc. (Tokyo, Japan) for this research, which enables communication from the island coast to approximately 20 km offshore in the experimental sea area of the Yaeyama Islands (https://www.docomo.ne.jp/area/).

### Testing the logger on sea turtles

#### Developed soundscape logger

**Figure 4:**
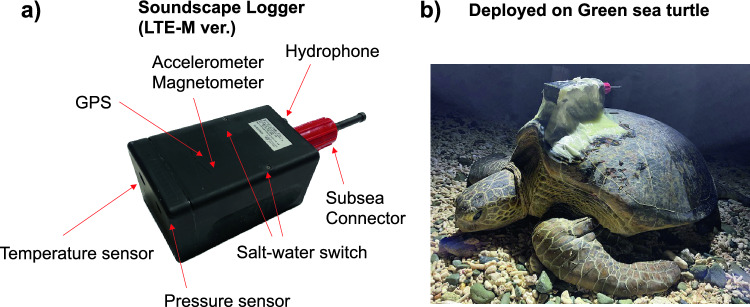
Soundscape logger and the deployment to a sea turtle. (**a**) A photograph illustrating the developed data logger, and (**b**) the data logger deployed on a green sea turtle (*Chelonia mydas*).

The developed data logger measures 160 mm in length, 100 mm in width, and 85 mm in height, excluding a connector and hydrophone. It weighs 2 kg in the air and approximately 0.6 kg in the water (Fig. 4a). It is equipped with a 3-axis accelerometer, a 3-axis magnetometer, a depth sensor, a temperature sensor, and a GPS. The accelerometer, magnetometer, depth sensor, and temperature sensor record data every 60 s, while the GPS operates similarly to the FastLoc GPS^40^, which is widely utilized in marine animal tracking and enables fast positioning when sea turtles surface. In a preliminary accuracy evaluation test conducted with sea turtles in a 10 m × 10 m outdoor tank, we obtained a total of 1500 location data points from three loggers used in the test. The root mean square error (RMSE) for these data points, representing the deviation from the turtles' nearly fixed latitude and longitude within the tank, was 32.04 ± 26.8 m. An AS-1 hydrophone (Aquarian Hydrophones, WA, USA) was utilized, featuring an omnidirectional design with a flat sensitivity response of − 208 (± 2) dB re: V/μPa from 1 Hz to 100 kHz. Audio data was sampled at 44.1 kHz with a 16-bit resolution, and recordings were made at 30-min intervals for 5 min (300 s) in a duty cycle. The 5-min audio data were subsequently processed internally within the logger as follows.

1. Divide the 300-s data into sixty 5-s segments.
2. Compute a spectrogram (with 0.5 FFT overlap) for each 5-s segment and apply a median filter.
3. Overlap and slide the resulting 5-s spectrogram data by 1 s within a 2-s time window, generating four images (192 × 192 pixels) per 2-s time window.
4. Perform image classification on each image using the pre-built TensorFlow Lite MobileNet ver. 1 model, with the outcome as the audio classification result.

The processing flow described above generates 240 images from the 5-min data, with each image representing a distinct sound, yielding 240 classification results, one for each sound. Although it is possible to transmit all classification results if continuous stable communication is available, when attached to a sea turtle, communication can only occur when the turtle surfaces to breathe and the logger, affixed to its carapace, is exposed to the air. Therefore, to reduce the data size, summarized data were sent. Various data summarization methods could be considered, but in this case, we assumed that the probability of each class from the 240 classification results was averaged over the 240. The class with the highest probability in its top three was used as the data for a single sound classification result. This summary is considered the most dominant sound classification result within the 5-min interval. One set of data for a single classification result was further combined with depth, temperature, vectorized acceleration (the square root of the sum of the squares of the three-axis acceleration components), and vectorized magnetic data (the square root of the sum of the squares of the three-axis magnetic components) measured at the beginning of the 5 min during the sound measurement. The vectorized acceleration and magnetic data, along with the depth and temperature, are crucial for understanding the activity level^53^ and geolocation^54^ of the animal. These metrics are essential for comprehensively monitoring the animal's behavior and movements, and therefore, are measured and transmitted along with the sound classification data.

The logger also has a saltwater switch to detect when a sea turtle surfaces, allowing for GPS measurements and data transmission via LTE-M. GPS measurements are taken each time a sea turtle surfaces. Each transmission packet can store up to 13 classification or position values. The logger initiates transmission each time it becomes feasible, prioritizing previously unsent data chronologically. If three consecutive transmission attempts fail, the data is discarded, and the subsequent new data is transmitted. Although the saltwater switch enables the logger to prepare for transmission, it requires at least 6–10 s for the LTE-M transmission function to activate and establish communication. Transmission may fail due to sea turtle behavior or wave conditions. As green turtles remain on the surface for approximately 10 s per breath (see, for example, the post-dive surfacing duration in Okuyama et al.^55^), it was considered achievable for communication to occur despite the relatively long activation time.

With the above settings, the logger is equipped with a 30,000 mAh lithium polymer battery. It is designed to operate for 40 consecutive days under specific conditions: the accelerometer, magnetometer, depth sensor, and temperature sensor record data every 60 s, and recordings are made for 5 min at 30-min intervals in a duty cycle. This estimate assumes no transmission, as the transmission frequency and power consumption greatly depend on the sea turtle’s breathing rate and the local signal environment. Since the timing of sound classification and GPS positioning differ, the most recent GPS location within an hour before or after the sound classification was used as the location where the sound classification occurred. If the loggers can be retrieved directly, the unaggregated classification results and all GPS position data, 3-axis accelerometer, 3-axis magnetometer, depth sensor, and temperature sensor data can be downloaded.

#### Field trials using sea turtles

To validate the practical application of the developed loggers for field data collection, they were attached to nesting green sea turtles (*Chelonia mydas*). Surveys were conducted at Ibaruma Beach, Ishigaki Island, Okinawa Prefecture, Japan (24°20′ N, 123°50′ E) during the summer of 2022. This site has been continuously monitored in previous studies^56^, and the logger attachment procedures were consistent with those used in past research (Okuyama et al., unpublished data). Briefly detailing the process, after they finished oviposition, we measured the straight carapace length and width (SCL and SCW) and the body weight, cleaned the carapace with sandpaper, and attached the tags securely to the carapace using epoxy resin (Konishi Co., Ltd. Osaka, Japan) and fiberglass cloth (Tokyo Garasu Kikai Co., Ltd. Tokyo, Japan) (Fig. 4b). After the resin had completely dried, the turtles were released into the sea.

Sea turtles that come ashore to lay eggs do so multiple times within a single nesting season, allowing for direct logger collection. The loggers were designed to transmit data via LTE-M communication when the turtles surfaced; however, since not all data could be transmitted, logger retrieval was conducted whenever possible during subsequent nesting events.

Despite the relative heft of our loggers, which were 2 kg in air, their weight comprised less than 3% of the average body weight of the nesting green sea turtles we studied. These turtles, mature females coming ashore for nesting, had an average straight carapace length (SCL) of about 100 cm, aligning with previous studies on the same nesting beach that reported an average weight of 131 kg (based on measurements of 120–153 kg, average 131 kg, N = 6; Okuyama et al., unpublished data and Obe et al.^57^). This proportion falls comfortably within the traditionally recommended limit of 3–5% of body weight for attachments^58^, a guideline designed to minimize behavioral impacts. We took care to select turtles without limb injuries or deformities for logger attachment, and all loggers were successfully retrieved after observing the turtles’ normal nesting behavior on their return. The loggers and epoxy were completely removed at the time of recovery.

Analyses were performed regarding data recovery rates, the composition of acquired sound species, and the relationships among sound species, location, and depth data (acceleration, magnetism, and temperature data were not analyzed in this study). The data recording period was calculated from the difference between the first and last recording date and time of the data recovered from the internal memory of the recaptured loggers. For sound classification results combined with location data that yielded a relatively large number of points (e.g., more than 10 points) per sound source (e.g., Giant moray *Gymnothorax javanicus*; see “Results” for details), a kernel density distribution (a nonparametric method used to estimate data distribution) was calculated as an example of how sound species distribution (soundscape maps) could be represented. All analyses were performed using Python.

### Ethics statement

The study was carried out in compliance with the ARRIVE guidelines. All turtle handling procedures were conducted under special supplemental permits obtained from the Ministry of the Environment and Okinawa Prefecture (Ministry of the Environment permit number 2207282, Okinawa Prefecture permit number 4-2), and ethics approval from the Animal Experimentation Committee of the Fisheries Technology Institute (YA2022-03).

## Results

### Sound classification performance

A fully quantized TensorFlowLite model, based on the MobileNet ver. 1, was developed using the training image data, resulting in a 370 kByte model that could fit within the RAM size of the microcontroller. Simulations with the created model demonstrated Top1 accuracy rates of 91.3% for Level 1, 86.7% for Level 2, and 62.3% for Level 3. The accuracy rates for the Top 5 model were 97.1% for Level 1, 96.3% for Level 2, and 86.3% for Level 3. Recall, Precision, and F-measures are presented in Table 1. To provide a comprehensive assessment of our model’s performance across all classes, the confusion matrices for each classification level (Level 1, Level 2, Level 3) are illustrated in Supplementary Figs. S1, S2, and S3, offering insights into the model’s accuracy for each category.

**Table 1:** Classification results for 52 sound types using the TensorFlowLite model.

Top1		Recall (%)	Precision (%)	F-measure (%)
	Level 1	85.8	82.1	82.2
	Level 2	73.1	75	65.1
	Level 3	43.7	52.7	41.8

Top5		Recall (%)	Precision (%)	F-measure (%)
	Level 1	95.4	92.5	93.7
	Level 2	95	89.6	91.4
	Level 3	74.5	85.4	75.0

Refer to Supplementary Table 1 for classes at each level.

### Field trials using sea turtles

In total, data for more than 110 h were obtained from seven individuals (Table 2). Although two out of the seven individuals experienced communication problems with the loggers, preventing any data recovery via LTE-M communication, a considerable amount of sound classification and sensor measurement data (153 points) and position data (778 points) were obtained from the other five turtles through LTE-M communication (Table 2). Furthermore, data were successfully retrieved from all seven individuals upon recapture. With an average measurement period of less than one day (Table 2), the expected continuous operation in terms of power was not attained; however, the recovery of sound classification results, sensor measurement results, and location data was achieved. We could recover sound classification and sensor measurement results (240 points) and position data (1092 points) for the seven directly recovered individuals. Among the five individuals with at least one successful LTE-M communication, 91.6% of the measured data for sound classification results and sensor measurements, and 95.3% for location data, were retrieved through LTE-M communication.

**Table 2:** Size of sea turtles fitted with loggers, duration of data recording obtained from the attached loggers, number of data points recovered via LTE-M communication, and number of data points retrieved directly from the logger’s internal memory.

Turtle ID	SCL*^1^ (cm)	SCW*^2^ (cm)	Recording period (h)	Via LTE-M communication		Via direct download	
				No. of position	No. of sound classification	No. of position	No. of sound classification
2	97.2	69.9	24.33	270	48	270	54
4	97.2	69.9	3.73	26	8	27	8
9	98.8	76.4	22.82	239	40	269	46
10	101.0	81.2	7.50	35	13	42	15
11	97.9	74.8	12.58	0	0	88	26
12	97.2	77.4	22.12	208	44	208	44
13	91.8	69.8	23.18	0	0	188	47
Sum			116.3	778	153	1092	240
Average	97.3	74.2	16.6	111.1	21.9	156.0	34.3
Std	2.8	4.5	8.5	121.6	21.3	103.1	17.9

Sound classification data is transmitted along with the sensor measurement data (i.e., depth, temperature, acceleration, and geomagnetism).

*^1^ SCL, straight carapace length, *^2^ SCW, straight carapace width.

The relationship between depth, location, and classification results was summarized using data from the loggers of seven directly collected individuals. The percentage of sounds classified as background noise was 76.4 ± 15.8%, accounting for more than 70% of the total. Since all data from ID4 was background noise, this individual was excluded from the subsequent analysis. The following analysis is based on the results from the remaining six individuals.

**Figure 5:**
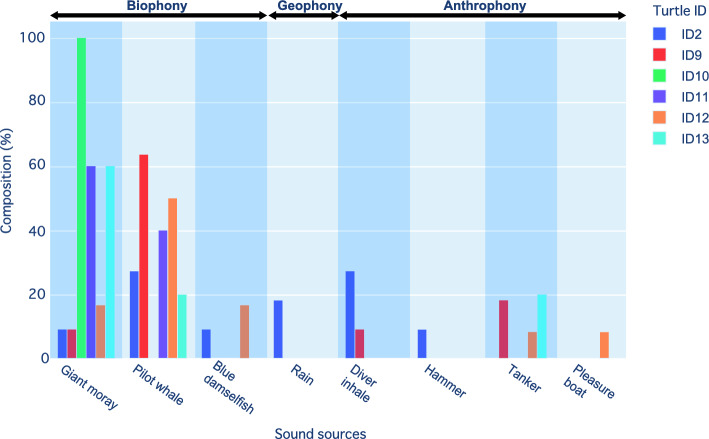
Sound source classifications from the field observations. Composition of sound source classifications obtained from each individual (6 individuals). This figure represents data collected at various depths. Further explanation regarding the use of depth-related data is provided in the “Results” section.

**Figure 6:**
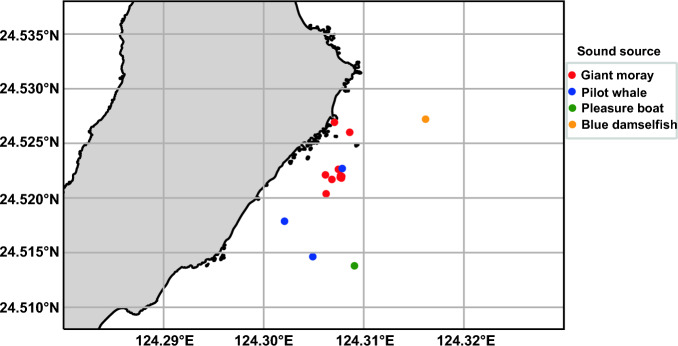
Soundscape map. Soundscape map: map of classified sounds and observed positions obtained from loggers (six individuals). This map primarily displays biophony, as no data were available for anthrophony and geophony when combined with the location data.

The composition of the sound classification results is displayed in Fig. 5. The composition for the six individuals was primarily composed of the Giant moray *Gymnothorax javanicus* (39.0 ± 40.2%) and the short-finned pilot whales *Globicephala macrorhynchus* (33.5 ± 22.7%), which had higher average percentages among the six individuals. Blue damselfish *Chrysipitera cyanea* was also observed in the composition. Although short-finned pilot whales are present in the field waters, the relatively high frequency of observations at shallow depths may suggest potential inaccuracies in the data. In the case of coral reef areas near the sea surface, the influence of currents and waves can also be assumed. Examining the sea turtle depth data, 73.7% of the sound observations were made when the experienced depth was shallower than 2 m (Supplementary Fig. S4). However, it is unclear whether the sea turtles were on the bottom or not due to the lack of data on the exact water depth at the site; we only extracted data when the experienced depth was 2 m or deeper, as currents and waves on the sea surface in coral reef areas close to the surface may affect errors in sound classification. In this case, the number of short-finned pilot whales substantially decreased from 17 to 3, while the number of Giant morays remained almost the same, decreasing from 11 to 10. By combining the location data and sound classification results with the data extracted only at depths greater than 2 m, a soundscape map could be generated (Fig. 6). For the Giant moray, the kernel density distribution (Supplementary Fig. S5) was drawn from the observation points as an example.

## Discussions

The logger developed in this study is considered the world's first biologging device system that employs deep learning for sound source classification and transmits the data when organisms surface. This innovative approach has the potential to greatly enhance our understanding of marine ecosystems by providing detailed insights into the acoustic activities of various organisms. With the integration of positional data, the capability to generate a kernel density distribution has been suggested by the example of the Giant moray in the results. However, we recognize that these representations are preliminary and require further validation to confirm their accuracy in depicting organism distributions. The implications of this technology extend to various aspects of biophony, anthrophony, and geophony. Moving forward, it is expected that multi-point and long-term observations through biologging are expected to facilitate the creation of detailed soundscape maps that accurately reflect the distribution of various sound sources.

Our study demonstrates that animals can serve as autonomous mobile platforms for collecting soundscape information in addition to oceanographic data. Real-time monitoring of soundscapes, including source-specific information, can enhance our understanding of sound diversity and enable more targeted actions when addressing conflicts between animals and human activities. The system is expected to be helpful in areas where buoy installation is challenging. A similar approach involving gliders has been employed^19,20^, but gliders are often challenging to operate in shallow coastal areas. Previous studies aimed to detect particular marine mammal sounds, such as those from whales and dolphins, rather than identifying multiple sound sources in biophony, anthrophony, and geophony, as in this study. An example of sea turtles being used for sound observations exists^59^; however, the study focused on measuring sea turtles' escape behavior in response to ship sounds rather than soundscape observations.

In this study, a microcontroller was selected as the hardware for sound processing. Implementing the same pre-processing and deep learning model on a microcontroller as on a PC with ample computational resources is challenging. However, we demonstrated that a biologging device could be implemented by reducing the complexity of the model and pre-processing. The results for the level 3 classification showed that although the Top 1 accuracy is not high at approximately 60%, relatively high accuracy can be achieved by considering the Top 5 accuracy (more than 85%). The execution of pre-processing and models depends on the microcontroller’s performance. As the demand for AI at the edge continues to grow^27^, low-power-consumption microcontrollers with edge processing capabilities are being developed to meet this demand^60^. Consequently, more significant amounts of RAM and memory can be installed in microcontrollers, and more energy-efficient microcontrollers will become available in the future. At that point, it will be possible to further increase the pre-processing time window length and FFT overlap ratio using the present method. Additionally, deep learning models will be able to incorporate more complex, higher-accuracy models.

One advantage of utilizing a microcontroller in this study is the updatability of the deep learning model. Although the current model was constructed using 52 sound classes, in the future, as the number of sound sources and samples increases and a retrained model is developed, the model can be easily replaced. The fully quantized 8-bit model allows the current system to handle up to 256 sound classes. A dedicated AI chip could be employed for embedded applications; however, it would be costly to develop and require designing the chip to facilitate model replacement.

Regarding power-saving, more efficient methods can be considered. This study conducted sound observations for 5 min every 30 min. However, sea turtle behavior also influences the sounds observed during those 5 min. For example, sounds were recorded when sea turtles were at or near the surface (e.g., shallower than 2 m as in this study). During such times, the sound of the sea turtles' breathing and wave or current noise hinder accurate monitoring of the environmental sounds composing the soundscape. Therefore, while it is difficult to determine whether the sea turtles were on the bottom due to the lack of data on the exact water depth at the site, excluding recordings at shallow experienced depths would allow for avoiding unnecessary data collection.

In the future, it is essential to consider how to summarize and transmit the sound classification results obtained from the deep learning model. For depth and temperature data, previous studies^26,61^ have effectively reduced the amount of transmitted data by summarizing the behavior of target organisms. In this study, we calculated the average probability for each class based on 5 min of continuous classification and decided to transmit the top three classifications. Although the transmission design did not include the Top 5 data for each image classification, using the average probability of continuous classification over 5 min allows for obtaining classification results with a relatively high probability during that timeframe. This approach enables the assessment of the sounds comprising the average soundscape over 5 min but sounds that infrequently occur during the period would receive lower rankings and may not be captured.

It should be noted that our system may face challenges in accurately classifying sounds in two scenarios: when multiple sound sources overlap in a single spectrogram and when encountering sounds not present in the training database. This study made an effort to minimize the impact of overlapping sound sources by adopting a short time window of 2 s for sound recording; however, it should be noted that cases with overlapping sounds were not explicitly accounted for, as the sound library excludes recordings with such overlaps. When the system encounters either sounds not present in the training database or potential overlaps in the spectrogram, these sounds are likely to be classified into one of the nearest matching classes we have, which could lead to misclassification. The system calculates the probability of each spectrogram belonging to every class, resulting in a probability distribution across all the classes for every individual image. Although this method provides valuable insights, it has limitations in distinguishing overlapped sounds or recognizing novel ones not included in the training set.

Based on the classification results obtained during the field experiment, certain species commonly encountered by local fishermen (personal communication with Mr. Taira; this is not an experiment on human participants) and reported in literatures^62–64^ were observed, such as Moray eels and blue damselfish. However, the classification outcomes also showed certain biological sounds commonly detected with a higher frequency at shallow depths, such as pilot whale sound, thus highlighting the potential for misclassification. Although the sound classifier installed on the sea turtle logger is expected to capture a variety of sounds that would be experienced by sea turtles, sounds associated with sea turtle behavior, including breathing, swimming, and the noise generated from interactions with natural structures, were not explicitly included in the sound classification model used in this study. Consequently, such sounds may have been mistakenly classified as one of the 52 sound species.

To address the limitation of our current sound classification model, which does not include sounds directly associated with sea turtle behavior, developing a dedicated sound library representing the unique acoustic characteristics of sea turtles is crucial. Furthermore, it is important to note that within our project, we employed a hydrophone-integrated video logger (LoggLaw CAM) in a separate experiment within our project. This device was attached to sea turtles not only to capture sounds directly associated with their behavior, but also to monitor the ambient acoustic environment, including sounds produced by other marine organisms. The results of this experiment, which will provide valuable insights into the acoustic characteristics of sea turtle behavior, will be detailed in a forthcoming publication.

While uncertainties remain regarding the accuracy of field observations, the significance of this study lies in the successful remote collection of near-real-time sound classification data from sea turtles equipped with loggers incorporating edge processing and a deep learning model. In order to enhance the reliability of the classification system, it is essential to repeat the cycles of conducting field validation of classification performance, expanding the training data set, and developing a more robust model. Future plans include evaluating the reliability of data with playback experiments. However, the main focus of this research is the development of the hardware, pre-processing techniques, and algorithms, making a comprehensive reliability evaluation beyond the scope of this study. In practical applications, the updated model can be readily installed in the loggers, facilitating advancements in sound classification performances.

As an important aspect of our research, the loggers used in this study were initial prototypes, specifically designed to validate our device concept. In this phase, we primarily utilized cellular networks available in the coastal regions of Japan for monitoring sea turtles nesting on the shore. Recognizing that marine animals often venture into offshore areas beyond the reach of cellular networks, we also developed a logger variant capable of Argos satellite communication. This new model, while retaining all the key specifications of the original design, weighs approximately half as much as the original. It’s worth noting that the outcomes and insights gained from the use of the Argos communication system will be reported separately, highlighting its efficacy and potential in further research applications. Importantly, the Argos satellite communication system enables data collection even during the post-nesting migration of sea turtles, ensuring continuous monitoring as they move into open oceanic waters. The current trend towards smaller and more energy-efficient electronic components, as well as high-performance microcontrollers or chips tailored for edge AI^60,65^, has created an environment where loggers can be further miniaturized. Additionally, these advancements make it feasible to equip loggers with more complex deep learning models for enhanced edge processing capabilities. These advancements not only enhance our research but also pave the way for new practical applications.

### Supplementary Information

Supplementary Information.

## Data Availability

Data are available from the Dryad Digital Repository (https://datadryad.org/stash/share/MN3MhpzQVaoed-vXJtbYL1eByqstEDL7FSbTRICxDTQ).
