# Supplementary material for: Animal-borne soundscape logger as a system for edge classification of sound sources and data transmission for monitoring near-real-time underwater soundscape
Source: Sci Rep. 2024 Mar 16;14:6394. doi: 10.1038/s41598-024-56439-x (PMC10944488; doi:10.1038/s41598-024-56439-x)
Supplement: Supplementary file 1 — Supplementary Information. [file 41598_2024_56439_MOESM1_ESM.docx]

**Supplement**

**Supplementary Table S1**

The sound source classes, the categorized levels, and the number of samples utilized in this study. This study selected 52 sound classes consisting of biological, geographical, and anthropogenic sounds as target sounds to classify them at three levels of detail. The levels range from primary classification (Level 1) of biophony, geophony, or anthrophony, to more detailed classification (Level 2), such as distinguishing fish sounds from marine mammal sounds within biophony or vessel sounds from construction sounds within anthrophony. The most detailed classification (Level 3) specifies animal species, call types within the same species, vessel types (fishing boats, cargo ships, pleasure boats, etc.), and even construction types (pile driving or excavation).　It is important to note that background noise is included in the same category as geophony in this study. For detailed information on the recording method, please refer to Tanaka et al. (unpublished data).

**Supplementary Table S2**

RAM size required to calculate input time window length (sec) for different input sound time window lengths and various FFT overlap ratios. The numbers in the table are the required RAM size in Kbytes. The white areas in the table indicate configurations that can be accommodated within the microcontroller's RAM (up to 512 Kbytes). In contrast, the red areas represent configurations the microcontroller cannot support. For the developed logger, a 5-second time window length and a 0.5 overlap ratio (indicated by the red frame) were selected, as these values permit a relatively long window and a high overlap ratio.

**Supplementary Fig. S1**

Level 1 classification accuracy confusion matrix using the fully quantized TensorFlowLite model (a: Top1, b: Top5). The numbers in the matrix indicate the percentage of corresponding classification results normalized with respect to the true class (horizontal axis). This matrix represents the classification outcomes for input audio sources, where the spectrogram of each audio sample is computed on a logarithmic scale covering 0–20kHz on the vertical axis and spans 2 seconds on the horizontal axis, resulting in images of 192x192 pixels.

**Supplementary Fig. S2**

Level 2 classification accuracy confusion matrix using the fully quantized TensorFlowLite model (a: Top1, b: Top5). The numbers in the matrix indicate the percentage of corresponding classification results normalized with respect to the true class (horizontal axis). This matrix represents the classification outcomes for input audio sources, where the spectrogram of each audio sample is computed on a logarithmic scale covering 0–20kHz on the vertical axis and spans 2 seconds on the horizontal axis, resulting in images of 192x192 pixels.

**Supplementary Fig. S3**

Level 3 classification accuracy confusion matrix using the fully quantized TensorFlowLite model (a: Top1, b: Top5). The numbers in the matrix indicate the percentage of corresponding classification results normalized with respect to the true class (horizontal axis). Please enlarge a separate PDF for a larger view. This matrix represents the classification outcomes for input audio sources, where the spectrogram of each audio sample is computed on a logarithmic scale covering 0–20kHz on the vertical axis and spans 2 seconds on the horizontal axis, resulting in images of 192x192 pixels.

**Supplementary Fig. S4**

Histogram of depth data from recovered loggers of six individuals combined.

*
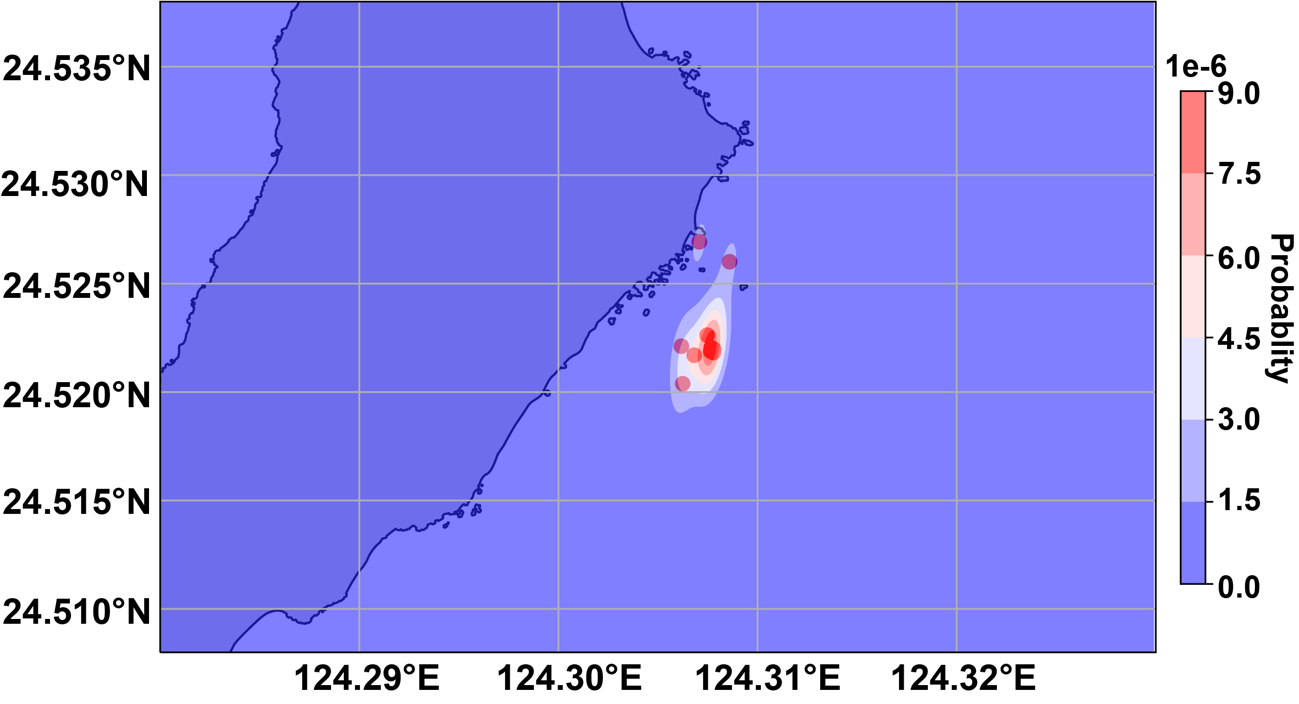
*

**Supplementary Fig. S5**

Kernel density distribution calculated from the observation positions for the Giant moray *Gymnothorax javanicus*.
